# Evaluation of community-based vector surveillance system for routine entomological monitoring under low malaria vector densities and high bed net coverage in western Kenya

**DOI:** 10.1186/s12936-023-04629-9

**Published:** 2023-07-03

**Authors:** Bernard Abong’o, Michelle C. Stanton, Martin J. Donnelly, Eric Ochomo, Feiko O. ter Kuile, Aaron M. Samuels, Simon Kariuki, George Musula, Richard Oxborough, Stephen Munga, Steve J. Torr, John E. Gimnig

**Affiliations:** 1 Centre for Global Health Research, Kenya Medical Research Institute, PO Box 1578, 40100 Kisumu, Kenya; 2 Liverpool School of Tropical Medicine, Pembroke Place, Liverpool, L3 5QA UK; 3 Malaria Branch, Division of Parasitic Diseases and Malaria, Center for Global Health, Centers for Disease Control and Prevention, Kisumu, Kenya; 4 Malaria Branch, Division of Parasitic Diseases and Malaria, Center for Global Health, Centers for Disease Control and Prevention, Atlanta, GA 30333 USA; 5 PMI VectorLink Project, Abt Associates Inc, 6130 Executive Blvd, Rockville, MD 20852 USA

## Abstract

**Background:**

Entomological surveillance is traditionally conducted by supervised teams of trained technicians. However, it is expensive and limiting in the number of sites visited. Surveillance through community-based collectors (CBC) may be more cost-effective and sustainable for longitudinal entomological monitoring. This study evaluated the efficiency of CBCs in monitoring mosquito densities compared to quality-assured sampling conducted by experienced entomology technicians.

**Methods:**

Entomological surveillance employing CBCs was conducted in eighteen clusters of villages in western Kenya using indoor and outdoor CDC light traps and indoor Prokopack aspiration. Sixty houses in each cluster were enrolled and sampled once every month. Collected mosquitoes were initially identified to the genus level by CBCs, preserved in 70% ethanol and transferred to the laboratory every 2 weeks. Parallel, collections by experienced entomology field technicians were conducted monthly by indoor and outdoor CDC light traps and indoor Prokopack aspiration and served as a quality assurance of the CBCs.

**Results:**

Per collection, the CBCs collected 80% fewer *Anopheles gambiae sensu lato* (*s.l*.) [RR = 0.2; (95% CI 0.14–0.27)] and *Anopheles coustani* [RR = 0.2; (95% CI 0.06–0.53)] and 90% fewer *Anopheles funestus* [RR = 0.1; (95% CI 0.08–0.19)] by CDC light traps compared to the quality assured (QA) entomology teams. Significant positive correlations were however observed between the monthly collections by CBCs and QA teams for both *An. gambiae* and *An. funestus*. In paired identifications of pooled mosquitoes, the CBCs identified 4.3 times more *Anopheles* compared to experienced technicians. The cost per person-night was lower in the community-based sampling at $9.1 compared to $89.3 by QA per collection effort.

**Conclusion:**

Unsupervised community-based mosquito surveillance collected substantially fewer mosquitoes per trap-night compared to quality-assured collection by experienced field teams, while consistently overestimating the number of *Anopheles* mosquitoes during identification. However, the numbers collected were significantly correlated between the CBCs and the QA teams suggesting that trends observed by CBCs and QA teams were similar. Further studies are needed to evaluate whether adopting low-cost, devolved supervision with spot checks, coupled with remedial training of the CBCs, can improve community-based collections to be considered a cost-effective alternative to surveillance conducted by experienced entomological technicians.

**Supplementary Information:**

The online version contains supplementary material available at 10.1186/s12936-023-04629-9.

## Background

Monitoring of mosquito populations for density, species composition, sporozoite infection and insecticide resistance status is important for evaluating the effectiveness of malaria vector control strategies. Currently, long-lasting insecticidal nets (LLINs) and indoor residual spraying (IRS) are the main malaria vector control strategies. Both are applied indoors and affect mosquito populations by reducing population densities [1–3], species composition [4, 5] and potentially vector behaviour [3, 6–8]. An entomological evaluation framework is required to detect changes in these outcome measures as an assessment of vector control operations.

Traditionally, entomological surveillance has relied on well-trained, closely supervised, centrally managed monitoring teams. However, this approach is usually limited in geographic scope and frequency of sampling [9, 10] due to the high cost of teams of specialised entomologists and transport to field sites [10, 11]. With the increasing need for enhanced vector control [12] and dramatically altered vectorial systems, supervised vector surveillance is envisioned to become even more challenging and expensive, highlighting the need for a devolved surveillance system. Devolved systems that adopt cost-effective trapping methods for local monitoring by resident community-based teams represent a potentially cost-effective alternative [9–11]. This strategy is anticipated to be affordable and sustainable on a large scale [9, 11, 13], and allows for more intensive sampling of each community [9].

While community-based entomological monitoring is reported to be cost-effective [9, 10], previous studies evaluating its effectiveness have identified challenges that limit its validation and implementation. First, when implemented with conventional mosquito sampling tools, there have been concerns about the quality of unsupervised data collection [10]. Quality-assured, community-based mosquito collections have been observed to have low sensitivity per night of trapping compared to quality-assured field entomology teams [9, 10]. Second, independent validation of unsupervised data collection has also proved challenging if community-based teams are aware of the supervision and can alter their behaviour during supervisory visits [9]. Lastly, implementation of community surveillance either requires simplified tools such as CDC light traps, which require batteries to be charged regularly and may be unfeasible in rural settings, or rely on alternative methods that may need validation before they can be widely implemented [9, 14].

This study aimed to implement and evaluate a community-based vector surveillance system for routine entomological monitoring in an area of low malaria vector densities and high LLIN coverage in western Kenya. The quality of the community-based sampling data for estimating vector species composition and seasonality was compared to sampling by experienced field entomology teams.

## Methods

**Fig. 1:**
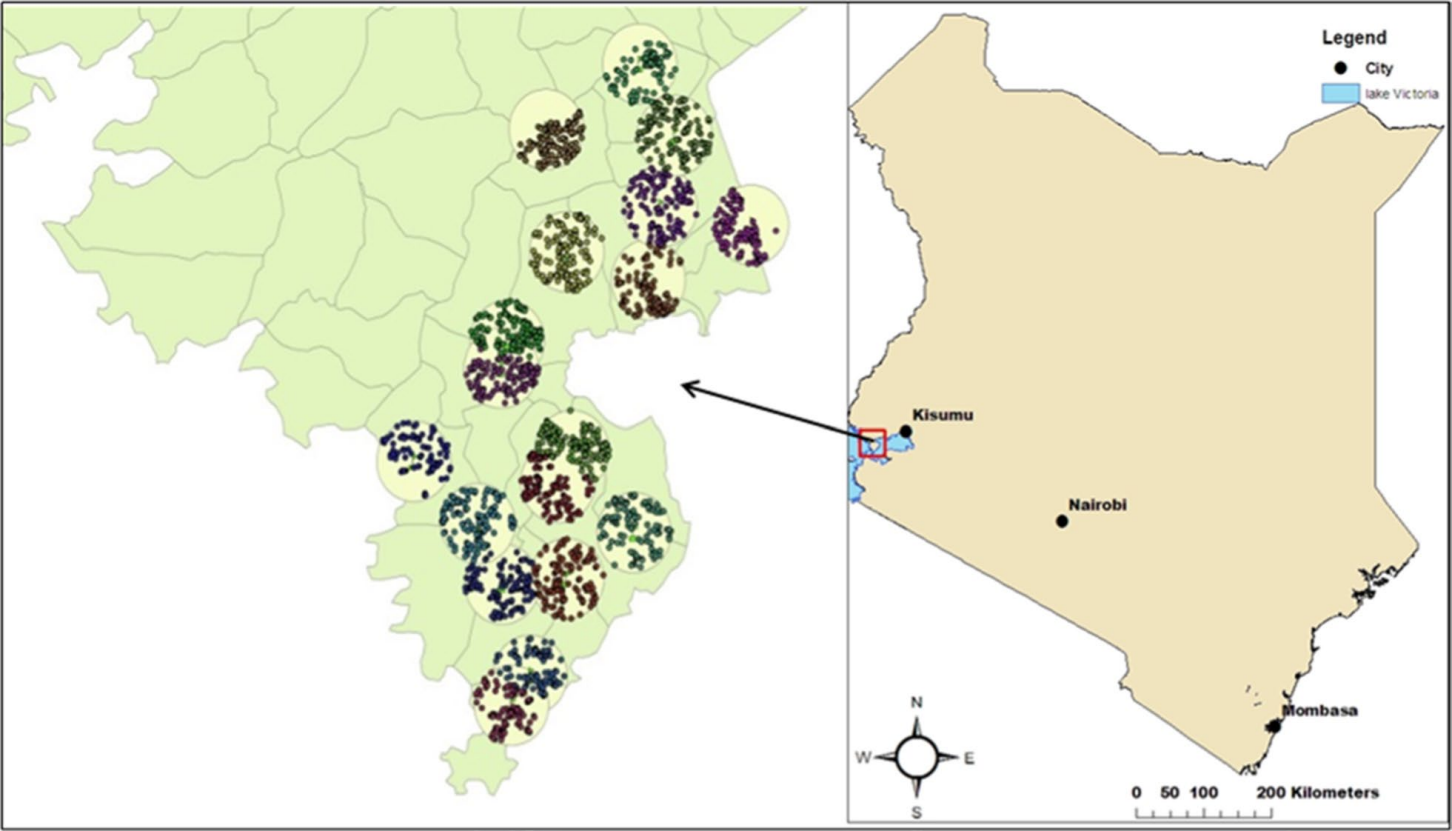
A map of Kenya showing the study area with the clusters and randomly selected compounds

A community-based vector surveillance system was implemented in the Asembo (−0.18139; 34.38552) and Uyoma (−0.31667; 34.3167) communities of Rarieda sub-county, Siaya County in western Kenya (Fig. 1). The study site is part of the Kenya Medical Research Institute (KEMRI) and Centers for Disease Control and Prevention (CDC) Health and Demographic Surveillance System, where the prevalence of microscopy-confirmed malaria among children < 5 years of age is 39% [15]. Malaria vector species in this region are *Anopheles funestus*, *Anopheles arabiensis* and *Anopheles gambiae sensu stricto* (*s.s.*) [16, 17]. The region has a bimodal rainfall pattern, with ‘long’ rains between April and June and ‘short’ rains in October and November, which are associated with increased malaria transmission.

### Study design

Eighteen clusters were designated in the study area, with each cluster ≈ 4 km in diameter and centred on the house of the collector. In each cluster, 60 houses were randomly selected for mosquito collection (Fig. 1). Each of the 60 houses was sampled monthly by indoor and outdoor light traps and indoor Prokopack aspiration between May 2017 and July 2018. Each CBC began their collections from a randomly selected reference house in each cluster and sampled a set of 3 neighbouring houses each day for 20 days. Each primary collection house was assigned two replacement houses for mosquito collection if the primary houses became unavailable.

### Selection and training of community-based collectors

A mixed approach was used in the identification and recruitment of the CBCs with the following inclusion criteria: (i) a resident of the community, (ii) resident of a house with a tin roof for installation of solar panels; (iii) ownership of a personal means of transport, preferably a bicycle or a motorbike, (iv) prior experience as either a community health volunteer (CHV) or mosquito collector, and (v) ability to operate a mobile device for data collection and transmission. The CBCs were identified through local health facilities if community health volunteers (CHVs) were recruited or through the local administrative authorities where the CHVs were unavailable. Of the eighteen collectors, six had prior entomological experience from previous studies in the region while the rest did not.

The CBCs were trained in mosquito collection techniques using CDC light traps and Prokopack aspiration. Additional training included basic mosquito identification using morphological features to differentiate between anopheline and culicine mosquitoes and between female and male mosquitoes, and to classify the physiological status of mosquitoes as either fed, unfed, gravid, or half-gravid. The collectors were also trained on the capture and transmission of entomological and household data using Open Data Kit (ODK) software on an Android mobile device. Other training included operating a solar charging system for charging light trap batteries and tablets, administering a questionnaire and the consenting process. All training included practical demonstrations and field practice and was undertaken over 5 days before the start of the study. After the initiation of mosquito collection, support training was provided to the CBCs as needed.

### Building and installation of the solar charging system

**Fig. 2:**
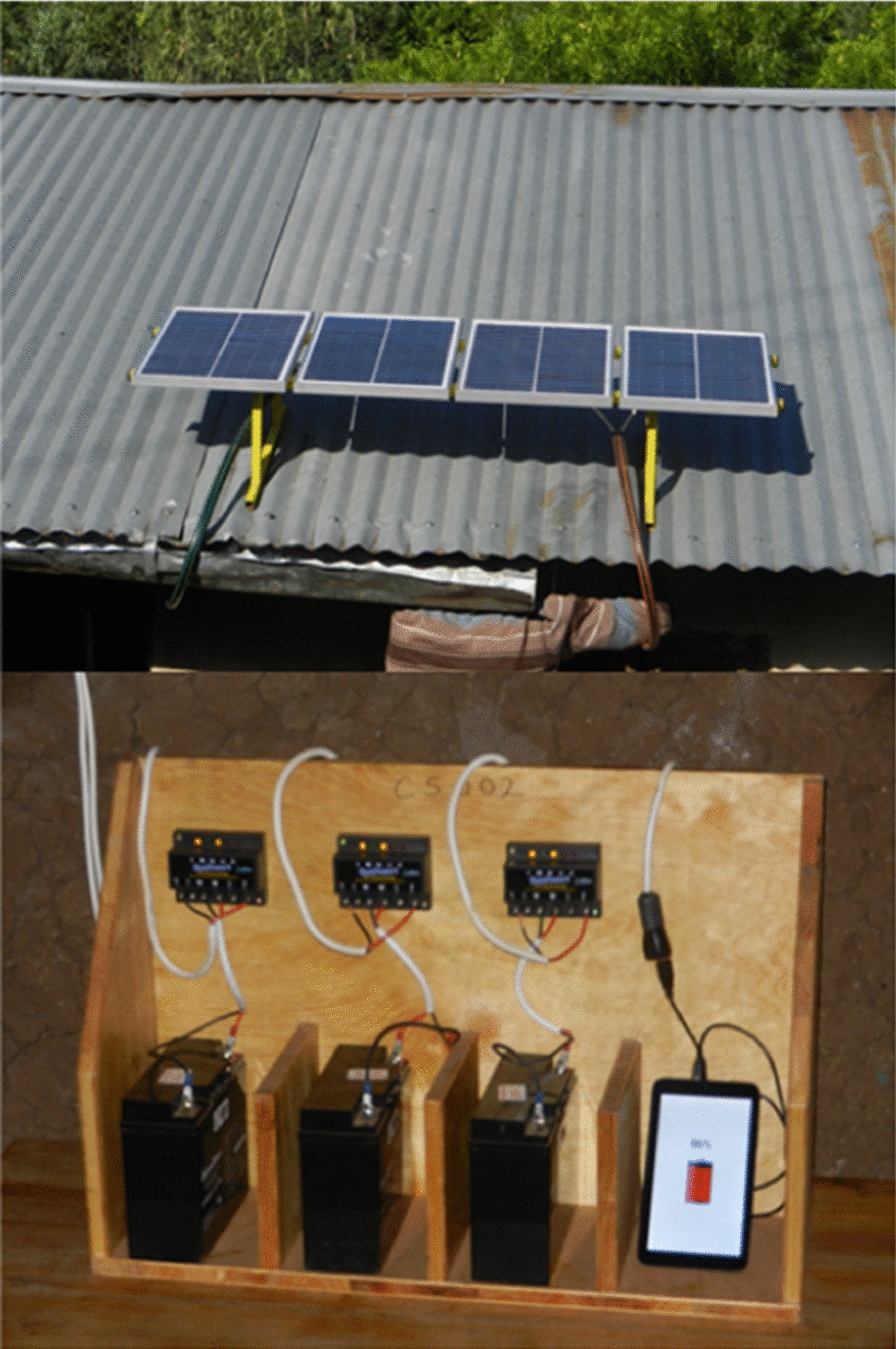
A picture of four solar panels attached to a metallic frame mounted on a roof and a charging station showing batteries connected to charge controllers and an Android tablet connected to a USB charging port

Eighteen solar charging units were assembled by a local engineer within Kisumu city (0.0917° S, 34.7680° E). Each unit was comprised of four solar panels attached to a lockable metallic frame. Three solar panels were connected to charge controllers (SolarTech^®^), with each charge controller connected to a 12 V rechargeable battery. The fourth solar panel was connected to a Universal Serial Bus (USB) cable for charging the tablet (Fig. 2).

During installation, the set of solar panels on a metallic frame was attached on top of a tin-roofed house belonging to the CBC. The frame was attached to the roof with screws from inside the house, while the screws holding the individual panels on the frame were blocked with a metallic plate to prevent theft. Cables from the solar panels were passed under the iron-sheet roof to connect to the charging station within the house.

### Equipment and material

Each CBC was issued with a solar charging unit, three 12 V rechargeable batteries with terminals modified to connect two light traps at the same time, six CDC light traps, three with 5 m long connecting cables for outdoor installation and three with 2 m cables for indoor trapping, a Prokopack aspirator and three collection cups for indoor mosquito collection, an adult mosquito cage, a mouth aspirator, three paper cups, a pair of forceps, a magnifying lens for mosquito identification and Petri dishes for transferring and holding collected mosquitoes. Other equipment included a tablet (Nexus 7, ASUSTek Computer Inc., Taipei, Taiwan) for the collection and transmission of data, data forms and consent forms. Additionally, a set of 20 mL scintillation vials with 70% ethanol for the preservation of collected mosquitoes was provided to the collectors bi-weekly and the tablets were loaded with data bundles for internet connectivity monthly.

### Consent

Before initial collections, under the supervision of project staff, the CBCs obtained written consent from the heads of all 60 randomly selected households in each cluster. After the initial written consent, verbal consent was sought from the household during each subsequent mosquito collection. Consent was only sought from the backup households when an original household withdrew from the study, and a replacement household was recruited for sampling.

### Mosquito collection

Mosquito collections by indoor/outdoor CDC light trap and indoor Prokopack aspiration were conducted in three houses per night. CBCs undertook collections over five consecutive nights each week, sampling each of the 60 consented houses monthly. Indoor CDC light traps were set in the sleeping area next to an occupied bed net at about 1.5 m from the floor. Outdoor traps were placed within 5 m from the house, suspended at 1.5 m from the ground on either a tree, pole or under the roof. Both traps were run from a single 12 V battery. The traps were operated from 18:00 h to 07:00 h the following morning. After removing the light traps in the morning, the Prokopak aspirator was used to collect any mosquitoes resting in the house.

During the mosquito collection period, the collector administered a brief questionnaire to collect information on household characteristics, including roof type, wall type, presence of eaves, presence of bed nets and net use, presence of cattle and number of people that slept in the house at every collection. The location of each house was recorded using a Global Positioning System (GPS) at each visit.

### Mosquito processing

The CBCs processed the mosquitoes in their homes by first shaking the collection cups until all the mosquitoes were knocked down. Magnifying lens was then used to assist in identification. The CBCs sorted mosquitoes by subfamily (anopheline or culicine), by sex, and by abdominal status (fed, unfed, gravid, or half-gravid). The number of mosquitoes in each of these categories was recorded on a paper form, and the data were subsequently entered into the tablet and transmitted to the cloud server. All mosquitoes were preserved together in 70% ethanol in a scintillation vial. Each vial was labelled with the collection date and method, and house code. The collectors were instructed to record and preserve any insect which they thought to be a mosquito.

The preserved mosquitoes and completed paper forms were collected from the field every 2 weeks and preserved mosquitoes underwent further processing at the KEMRI laboratories in Kisumu. Trained entomology technicians who were blinded to the results of the CBC identifications repeated the classification process performed by the CBCs. In addition, all mosquitoes of the *Anopheles* genus were further identified by the trained technicians to species/complex level using morphological features [18].

#### Monitoring of light trap battery charging cycles

The light trap batteries were charged daily, and the charge status was recorded at the beginning and end of every charging session. CBCs scanned the battery barcode label and recorded whether the battery was fully charged, half-charged or completely discharged using light indicators on the charge controllers. In addition, the CBCs submitted daily status reports on battery charge levels, any faults in the solar charging unit, lost/broken items, and needed supplies.

### Supervision of CBCs

Initial trainings on light trap collections and mosquito processing were provided within the first month of mosquito collection. Subsequent collections, sample processing and identification, were not supervised routinely. Monitoring of the collections was planned to be conducted remotely by daily reviews of submitted data, but because of budget limitations, data quality monitoring was rarely done. Cases of delayed or failed data submission were followed up directly with the individual CBCs, and where necessary, field visits were conducted to assist with specific challenges. Every CBC was visited fortnightly to pick up samples and provide new tubes with 70% ethanol. Specialized technical assistance with the solar charging system was provided routinely by a field-based technician within the first year of study. After the initial training at the beginning of the study, no additional structured training sessions were offered for the collectors, but technical support was provided as needed during regular supervisory field visits.

### Quality-assured collections by trained entomology technicians

Parallel collections by trained entomology technicians were conducted in eight of the eighteen clusters sampled by the CBCs for 15 months (May 2017 to July 2018) as a quality assurance check on the CBCs. The eight clusters were selected based on mosquito densities from the CBCs: three clusters with higher densities, three with the lowest and two with median mosquito numbers. The collections were conducted in the same houses as the CBCs within 2 weeks following the CBCs’ visit, without the CBCs’ knowledge. If houses sampled by the CBCs were unavailable, the quality assurance (QA) team selected a neighbouring house for sampling. The quality-assured collections were conducted for 1 week each month by indoor CDC light trap and indoor Prokopack aspiration in ten houses out of the 60 that were visited by CBCs each month in each of the eight clusters that were monitored by the QA team. The CDC light traps were deployed from 18:00 h to 07:00 h the following morning in the sleeping area next to an occupied bed. After the removal of light traps in the morning, indoor resting collections were performed by indoor aspirators in the same houses.

### Cost-effective analysis

The cost of each sampling scheme was estimated for indoor CDC light trap collections. The cost per sampling effort was approximated based on the total expenditures, including procurement and installation of equipment, personnel and transportation costs.

### Laboratory analysis

All mosquitoes were transported to the laboratory and identified to species level morphologically [18, 19], and the abdominal status was scored as either fed, unfed, gravid or half gravid. Female mosquitoes were divided into three parts; the head and thorax were used for determination of sporozoite infection rate by enzyme-linked immunosorbent assay (ELISA) [20], the abdomens of blood-fed females were kept for blood-meal host determination and the remainder of the specimen was used in polymerase chain reaction (PCR) analysis to identify species within the *An. gambiae sensu lato* (*s.l*.) [21] and the *An. funestus* group [22] and for future molecular genetic analysis. Approximately 30% of the mosquitoes morphologically identified as *An. gambiae s.l.* were randomly selected and identified to species by PCR. For the *An. funestus* group, only 20% were identified by PCR [22], as previous studies in this area had found that the only member of the Funestus group present in adult collections was *An. funestus s.s*.

### Data management and analysis

Data collection was undertaken using ODK Collect, designed with an interface to limit entry errors. The house code was unique for every house sampled, and each collection effort was uniquely identified by a combination of house code, collection method, and collection date. At the end of each collection, each collection cup, paper cup or light trap bag containing samples was labelled with a combination of variables to distinguish between different collections. The combination of date, collection method, and house code was used to track the samples through laboratory processing.

During morphological identification of the mosquitoes, a unique barcode was given to individual mosquitoes and used to link the various laboratory procedures to the individual mosquito, including species identification by PCR, analysis of sporozoite infection by ELISA procedure and blood meal analysis.

Data analysis was performed using R version 3.4.1. Comparisons of the number of mosquitoes collected for 15 months were made between the CBCs based on identifications by the trained technicians and separate collections by the QA teams from the same houses as the CBCs, approximately 2 weeks apart. Houses that were visited only by CBCs or QAs were excluded from the analysis. Mean abundance of *An. gambiae* complex, *An. funestus* and *Anopheles coustani* were calculated per trapping night for indoor CDC light trap and Prokopack aspiration, and a Generalized Linear Mixed Model (GLMM) was fitted to the data to measure the differences between the number collected by the different teams (CBCs vs. QA teams). Since the data were over-dispersed, a negative binomial model was fitted using the glmmTMB package in R. Collection team, and collection method were included in the model as fixed effects, whereas house was treated as a random effect. Subsequent analyses were done by negative binomial regression after aggregating the total number of mosquitoes collected per month and cluster. A test of association between CBC and QA collection was performed by Pearson’s correlation and Spearman’s rank correlation.

Although paired identifications of individual mosquitoes by CBCs and trained technicians were unavailable, comparing aggregated numbers of *Anopheles* mosquitoes from the same collections was done to assess the accuracy of identification by the CBCs. To assess the accuracy of identification by the CBCs, mosquitoes classified as *Anopheles* by each CBC were aggregated by collection date, house of collection, method of collection and the relative numbers identified in each collection (total *Anopheles* by CBCs/total *Anopheles* by laboratory technicians) was calculated.

## Results

### Comparison of CBCs and QA teams

A total of 14,563 *Anopheles* were collected from 89,706 collection efforts conducted by the CBCs from all methods combined and were sent to the laboratory for morphological identification by expert entomology technicians. Of these, 6,149 (42%) were identified by the expert entomology technicians as *An. gambiae s.l*., 6,481 (45%) as *An. funestus*, 1,930 (13%) as *An. coustani* and 3 (0.02%) as other *Anopheles*.

The QA teams collected 1,332 *Anopheles* mosquitoes from 2,041 collections efforts by indoor CDC light traps and Prokopack aspiration. Of these, 774 (58.1%) were identified by the expert entomology technicians as *An. gambiae s.l*., 506 (38.0%) as *An. funestus*, 49 (3.7%) as *An. coustani* and 3 (0.2%) as other *Anopheles*.

Of the 6,149 *An. gambiae s.l.* collected by the CBCs and identified in the laboratory, PCR was conducted on 2,045 (31.6%) for species identification. Of these, 1,539 (75%) were identified as *An. arabiensis* and 506 (25%) as *An. gambiae s.s.* Of the 6,481 identified as *An. funestus* by the expert technicians, PCR was conducted on 1,399 (21.6%) and all were confirmed to be *An. funestus s.s.*

**Fig. 3:**
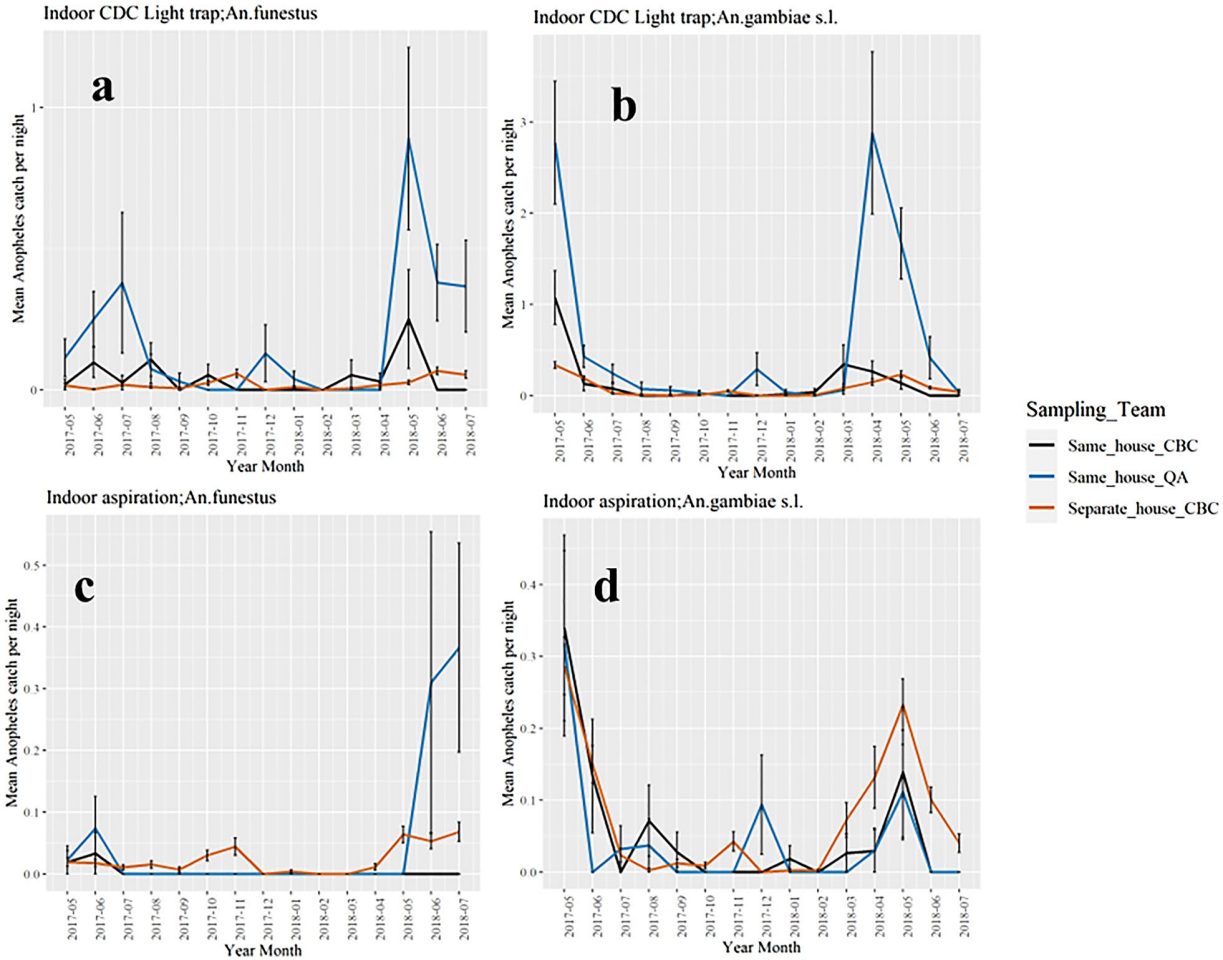
Monthly mean (means ± std errors) of *An. funestus* and *An. gambiae s.l.* by indoor CDC light trap and indoor Prokopack aspiration collected by trained entomology technicians (blue lines), community-based collectors in houses where trained entomology technicians also collected mosquitoes (black lines), and community-based collectors in villages where trained entomology technicians did not work (orange lines)

A total of 4,910 collections were conducted by both CBCs and QA teams in the same houses within 2 weeks over 15 months (May 2017 – July 2018), collecting 2,050 *Anopheles* mosquitoes. The QA teams made 1,024 collection efforts by indoor CDC light traps and 1,017 by Prokopack aspiration, while CBCs conducted 1,437 and 1,432 collections by indoor CDC light trap and Prokopack aspiration, respectively. Figure 3 compares the mean monthly catch of *Anopheles* species by indoor CDC light trap and Prokopack aspiration indoor between the CBCs and QA teams for collections performed in the same houses. The mean numbers of mosquitoes collected by CBCs in the 10 clusters not visited by the QA team are also presented. Overall, there were similar trends in the mean monthly densities of *An. funestus* and *An. gambiae s.l.* from indoor CDC light traps and indoor aspiration by CBC in clusters that had the quality-assured collections compared to those where no quality-assured collections occurred. The CBCs in clusters where QA collections were performed sampled significantly higher numbers of *An. funestus* and *An. gambiae s.l.* by CDC light traps and *An. coustani* by aspiration compared to CBC collections in villages where no QA sampling was performed. Statistical comparison of the mean *An. gambiae* and *An. funestus* catch by CDC light traps and indoor aspiration between CBC collections in clusters with QA teams compared to clusters where no quality-assured collections were performed are provided in Additional file 1: Table S1.

The mean number of *An. funestus* collected by indoor CDC light trap (Fig. 3) by the CBCs was low with no seasonal variation, while collections by the QA teams showed increased catch numbers between May and September. For *An. gambiae s.l* (Fig. 3b), April–June marked the period of high mosquito collection by both CBCs and QA teams, although the scales of the catches differed. The catch sizes during the rest of the months remained low. From Prokopack aspiration, the mean catch of *An. funestus* differed between the different sampling schemes between May and June, while the numbers remained low in the rest of the months (Fig. 3c). However, for *An. gambiae* s.l., the monthly catch sizes were comparable between the QA teams and the CBCs (Fig. 3d).

CBCs caught 80% fewer *An. gambiae s.l.* per collection effort compared to the QA teams, [RR = 0.2; (95% CI 0.13–0.38); P < 0.001]) by indoor CDC light traps. Similarly, the mean abundance of *An. funestus* collections by CBCs were 80% lower compared to QA teams [RR = 0.2; (95% CI 0.10–0.45); P < 0.001], while no significant difference was observed in the densities of *An. coustani* between the two sampling schemes. For indoor Prokopack aspiration, no significant difference in the mean catches of *An. gambiae s.l.* was observed between CBCs and the QA teams. However, significantly fewer *An. funestus* were collected by CBCs compared to the QA collections, [RR = 0.1; (95% CI 0.02–0.46); P = 0.01] (Table 1). By Prokopack aspiration, very few *An. coustani* were collected by the CBCs and none were collected by the QA teams.

**Table 1:** Comparison mean *An. gambiae* s*.l.* and *An. funestus* catch by indoor CDC light trap and Prokopack aspiration between CBCs and QA teams

Collection method	*Anopheles* species	Collectors	Efforts	Mean	RR (95% CI)
Indoor CDC light trap	*An. gambiae* s.l	CBCs	537	0.18	0.2 (0.14–0.27)^***^
		QA team	502	0.67	1
	*An. funestus*	CBCs	537	0.04	0.1 (0.08–0.19)^***^
		QA team	502	0.18	1
	*An. coustani*	CBCs	537	0.01	0.2 (0.06–0.53)^**^
		QA team	502	0.01	1
Prokopack aspiration	*An. gambiae s.l*	CBCs	531	0.06	1.3 (0.61–2.70)^NS^
		QA team	496	0.05	1
	*An. funestus*	CBCs	531	0.004	0.1 (0.02–0.46)^**^
		QA team	496	0.05	1
	*An. coustani*	CBCs	531	0.004	^−^
		QA team	496	0.00	–

*NS* Not significant

^**^P = 0.001

^***^p < 0.0001

When data were aggregated by month and cluster, the CBCs collected significantly fewer *An. funestus* by light trap compared to the QA teams [RR = 0.44, (95% CI 0.25–0.79); p = 0.006] though no difference was observed for *An. gambiae* [RR = 0.73; (95% CI 0.4–1.35); p = 0.318]. For Prokpack aspirations, the CBCs collected significantly more *An. gambiae* compared to the QA teams [RR = 5.22; (95% CI 2.43–11.2); <0.001]. No difference was observed in the number of *An. funestus* collected by Prokopack aspiration [RR = 0.85; (95% CI 0.36–2.01); 0.710]. A test of association between the CBC and QA collections by CDC light trap and Prokopack aspiration aggregated by month using both Pearson’s and Spearman’s correlation coefficients showed a significant positive correlation between the number of mosquitoes captured by CBCs and QA teams for both *An. funestus* and *An. gambiae* and for both light traps and Prokopack aspirations. The correlation coefficient values ranged from 0.01 to 0.62 for Pearson’s and from 0.19 to 0.36 for Spearman’s (Table 2).

**Table 2:** Pearson’s and Spearman’s correlation coefficients between CBC and QA collections of *An. funestus* and *An. gambiae* when aggregated by month and cluster

Species	Collection	Pearson		Spearman	
	Method	Rho	P-value	Rho	P-value
*An. gambiae*	Light trap	0.619	< 0.001	0.361	< 0.001
*An. gambiae*	Prokopack	0.201	0.037	0.361	< 0.001
*An. funestus*	Light trap	0.318	0.001	0.187	0.051
*An. funestus*	Prokopack	0.010	0.916	0.258	0.007
*Anopheles*	Light trap	0.620	< 0.001	0.351	< 0.001
*Anopheles*	Prokopack	0.072	0.459	0.275	0.004

### Cost effectiveness of CBC and parallel surveillance by supervised experienced team

All costs and exchange rates were estimated in 2017 in US dollars/Kenya shillings using an exchange rate of KES 100 per US Dollar. The QA collections were led by two technical staff plus a driver. Each technician supervised two experienced field assistants. The technical staff and the driver received their daily subsistence allowance of $70 (KES 7000) and $42 (KES 4200), respectively, during the period of travel. The time of the driver who picked samples from the CBC fortnightly was estimated at 0.1% of the entire salary cost. The other costs included casual payment for field assistants at a rate of $10 (KES 1000) per person per day, and transport and vehicle maintenance costs at $0.75 (KES 75) per kilometre covered. Equipment costs included the solar charging system $580.74 (KES 58,074), light traps $112 (KES 11,200) and tablets $113 (KES 13,000). Each CBC was paid a monthly stipend of $100 (KES 10,000). These costs may vary in other settings where different rates for staff travel allowance, casual payment, transport, and vehicle maintenance apply.

The cost per person-night was lower in the community-based sampling scheme compared to the collection by centrally managed teams of experienced, supervised collectors (Table 3). The cost of sampling by CBC was 9.8-fold lower compared to parallel sampling by the QA teams.

**Table 3:** Cost estimated per sampling scheme, per person night of sampling over fifteen months by both CBC and experienced parallel surveillance QA teams

Estimated parameter	Description	Number of units for CBCs	Number of units for supervised collections	Cost/Unit (KES)	CBCs $(KES)	QA teams $(KES)
Personnel costs	Entomology technician	1 for 15 months	2 for 15 months	175,000	26,250 (2,625,000)	52,500 (5,250,000)
	Driver	1 for 15 months	1 for 15 months	75,000	1,125 (112,5,00)	11,250 (1,125,000)
	Casual workers	–	4 for 15 months	5,000	–	3,000 (300,000)
	CBCs	8 for 15 months	–	10,000	12,000 (1,200,000)	–
Per diem costs	Entomology technician	–	2 for 15 months	35,000	–	10,500 (1,050,000)
	Driver	–	1 for 15 months	21,000	–	3,150 (315,000)
Equipment cost	Solar charging system	8 systems	–	58,074	4645.9 (464,592)	–
	Light traps	24	20	11,200	2,688 (268,800)	–
	Light trap batteries	–	20	5,353	–	1,070.6 (107,060)
	Tablets	8	2	13,000	1,040 (104,000)	-260 (26,000)
Transport and vehicle maintenance costs	Cost per kilometre	420 per month	660 km per month	75	4,725 (472,500)	7,425 (742,500)
Total expenditures					52,473.9 (5,247,390)	91,395.6 (9,139,560)
Number of sampling efforts					5735	1024
Cost per collection effort					9.1 (910)	89.3 (8,925.3)

### CBC identification accuracy

A total of 89,706 tubes with preserved mosquitoes, each representing a collection effort by CBCs, were submitted to the laboratory for identification by expert entomology technicians. While mosquitoes from all 89,706 tubes were identified and sorted by the trained entomology technicians, data on identification by the CBCs was only available for 10,256 (11%) of the tubes. This was due in part to the failure of some CBCs to upload their digital identification records to the cloud server and in part to a server crash that led to loss of the digital data on identification by the CBCs. However, the paired design of identifications by both CBCs and trained technicians allowed for a comparison of the relative accuracy of the CBC identification based on 10,256 collections. The CBCs identified a total of 11,088 male and female *Anopheles* mosquitoes compared to 1,840 that were identified by expert entomology technicians as *Anopheles*. Among the remaining insects, 3,847 were insects other than mosquitoes, while 5,401 were culicine species. Non-anopheline mosquitoes identified in each collection included primarily *Culex* mosquitoes but also smaller numbers of *Aedes* mosquitoes. The CBCs were more likely to inaccurately identify a mosquito or other insect as *Anopheles* than the trained technicians. The CBCs identified, on average, 0.93 mosquitoes as *Anopheles* per collection effort, whereas the expert technicians confirmed only 0.32 to be *Anopheles* per collection effort.

Across the entire collection period, the ratio of *Anopheles* identified by CBCs compared to the QA staff was 1.7 (1173/696) for experienced CBCs compared to 6.5 (5437/839) for those without any experience. This pattern was consistent across collection years as the CBCs without prior experience did not improve with practice compared to their counterparts with prior entomological experience.

## Discussion

Community-based collections using CDC light traps without supervision by professionals caught substantially fewer *Anopheles* mosquitoes per collection effort than quality-assured collections. Furthermore, compared to the QA teams, CBC collections showed limited seasonal variation, with monthly mean values consistently low across the year. When aggregated by cluster and month, the CBCs collected significantly fewer *An. funestus* by light trap despite approximately six times higher number of trap nights. However, the difference in the number of *An. gambiae* collected by the CBCs and the QA teams was not significant when aggregated. When aggregated by month and cluster, the CBCs collected significantly more *An. gambiae* compared to the QA collectors using Prokopack aspiration due to the larger number of collections. Furthermore, the aggregated numbers of *An. gambiae* and *An. funestus* collected by the CBCs was significantly and positively correlated with the numbers collected by the QA teams for both light traps and Prokopack aspirations suggesting the CBC collections may detect similar trends despite the lower number of mosquitoes collected per trap-night. These observations indicate that while the CBCs collected fewer mosquitoes per collection effort, the numbers collected were generally correlated with the numbers collected by the QA teams. If programmes only require overall trends in mosquito numbers, they may be able to utilize data aggregated by month using CBCs as the large number of collections may compensate for the low number collected per trap-night.

The observed differences in the number of *Anopheles* collected between the two sampling schemes suggest poor compliance by the CBCs. Furthermore, the CBCs in clusters where quality-assured sampling was conducted by expert entomology technicians collected greater numbers of mosquitoes compared to those who were not monitored. Although these comparisons are among CBCs working in different villages and may simply reflect geographic differences in mosquito densities, this observation raises the question of whether less supervision resulted in poorer compliance by the CBCs. In theory, the CBCs were unaware of the monitoring by the QA teams but in practice, it is suspected that the CBCs in the villages where the QA teams worked were aware of their presence as they were working in the same households. While direct observation was not possible and may have affected the performance of the CBCs, a number of reasons are suspected to have contributed to the low numbers collected by the CBCs relative to the supervised teams. One possible explanation is the setting of traps in improper locations by the CBCs. For best performance, the indoor light trap should be approximately 1.5 m from the floor, at the foot-side of an occupied bed net [23]. However, the installation of light traps in the sleeping area may be considered intrusive by some households and, at times, requires an explanation by the collector before consent is granted. It is possible that the CBCs might have failed to gain access to the sleeping areas and installed the traps in other rooms where no persons were sleeping under nets to attract mosquitoes. In addition, the CBCs experienced challenges arising from household fatigue due to repeated sampling from the same houses over time. While the recruitment of replacement houses addressed this challenge in cases where primary houses withdrew consent, some CBCs were found to have visited certain houses two to three times a month rather than once per month as instructed. Some collectors resorted to sampling repeatedly from more receptive households while avoiding those that resisted. Equipment failures also happened, with nearly all batteries requiring replacement after 1.5 years of use.

CBCs consistently misidentified *Anopheles* mosquito species from their collections. In attempts to distinguish between *Anopheles* and *Culex* species using morphological features, the teams reported more *Anopheles* than there were in each collection. A review of the morphological identification by a team of experienced entomology technicians observed over 75% fewer *Anopheles* mosquitoes compared to identification by CBCs. However, substantial variability in identification skills was observed between the CBCs. Those with previous entomological skills before being engaged as CBCs performed better at identifying *Anopheles*. However, the rate of misidentification by inexperienced CBCs did not improve throughout the study period. Additional training and possibly additional equipment, such as inexpensive dissecting scopes, may be required to improve the quality of identification by CBCs. Alternatively, programmes may need to continue relying on centralized mosquitoes’ processing.

Previous studies have highlighted the advantages and disadvantages of community-based mosquito sampling schemes. In a study in Tanzania, a community-based mosquito sampling scheme using Ifakara Tent Traps (ITT) was less sensitive compared to the gold standard human landing collection (HLC) but it was a much more cost-effective approach that enabled intensive surveillance of adult malaria vector populations [10]. Another study in Zambia reported that community-based collections using light traps were less sensitive than quality-assured collections but were cost-effective and broadly corresponded to malaria positivity rates as measured by active surveillance [9]. However, both studies identified challenges with validating data collected by community teams. In Zambia, it was suspected that collection by community-based teams improved during visits by supervised teams for quality-assured data collection due to prior knowledge of such visits [9]. To assess the quality of entomological surveillance in a setting with limited supervision, the community-based sampling scheme described here was implemented using the CDC light trap which has been previously reported to be effective for vector sampling in western Kenya [24]. Using a mobile-based data collection and transmission system allowed for remote monitoring of the CBCs and allowed the QA team to trace the houses sampled by CBCs without contact with the latter group. The use of light traps through a community-based surveillance system, validated by independent quality assurance data collection demonstrated the potential of unsupervised devolved entomological surveillance but also highlights the challenge of implementing such a surveillance system with limited supervision.

Community-based approach was observed to be substantially less expensive compared to the quality-assured sampling scheme as defined by the cost per collection effort. Previous studies comparing both sampling schemes have demonstrated the cost-effectiveness of community-based sampling approaches. A study in Zambia reported the cost of each sampling effort to be $289.40 and $13.60 for quality-assured and community-based light trap collections, respectively [9]. In Tanzania, the cost of sampling effort was approximately $55.90 and $4.80 for quality-assured and community-based Ifakara Tent Trap collections, respectively [10]. The costs per sampling effort was approximated to be $89.30 and $9.10 by quality-assured collections and community-based sampling schemes, respectively. Therefore, quality-assured collections by supervised teams cost 21.3 times more in the Zambian study, 11.6 times more in the Tanzanian study and 9.8 times more in our study per sampling effort compared to the community-based sampling. The reduced cost allowed for more intensive sampling at each site. While comparisons were made based on 10 houses sampled per cluster by the trained entomology technicians each month, the CBCs were able to sample 60 households in their assigned areas each month. The intensive sampling may provide more robust estimates of mosquito densities and reveal micro-geographic variation within villages. However, while the community-based sampling scheme was less expensive and enabled more intense sampling over a large geographical area, further improvements are required for optimization, which may increase the overall cost of this approach.

For future studies adopting a community-based sampling scheme, it may be useful to consider randomly selecting new houses periodically to avoid fatigue on the part of the households that may lead to refusal. Alternatively, where prior randomization of house is not possible, the collectors may be allowed to sample from all houses within the study site rather than restricting them to a set of a few selected houses, which limits their options in cases where consent is withdrawn. This would allow maximum coverage in terms of sampling households within the village and reduce study fatigue by participants, but it would also mean that different houses would be sampled each month. Also needed is an evaluation to see if a community-based sampling scheme with integrated low-cost, devolved supervision to provide spot checks can improve CBC compliance with light traps installation standards and support with challenges of any arising resistance in the community and improve quality. Additionally, routine remedial training of the CBCs on light trap installation and mosquito identification is recommended for improved performance.

## Conclusion

Unsupervised community-based mosquito surveillance by indoor CDC light traps substantially underestimated the mosquito population compared to the quality-assured collection by experienced entomology technicians. While the community-based sampling scheme was cost-effective with concurrent sampling in several locations, the CBCs captured significantly fewer mosquitoes per collection and misidentified a large number of mosquitoes and insects. Evaluating community-based sampling approaches with integrated devolved low-cost supervision with spot checks to enforce compliance and remedial training to improve mosquito identification skills is needed. The use of solar panels to charge light trap batteries and mobile data collection and transmission systems provides a sustainable system for routine, daily entomological monitoring in rural Africa.

## Supplementary Information

**Additional file 1.** Comparison of mean *An. gambiae s.l. and An. funestus* catch by CDC light traps and indoor aspiration between CBC collections in clusters with QA teams compared to clusters where no quality-assured collections were performed.

## Data Availability

Data is available at request from the corresponding author.
