# Supplementary material for: Evaluation of community-based vector surveillance system for routine entomological monitoring under low malaria vector densities and high bed net coverage in western Kenya
Source: Malar J. 2023 Jul 3;22:203. doi: 10.1186/s12936-023-04629-9 (PMC10318638; doi:10.1186/s12936-023-04629-9)
Supplement: Supplementary file 1 — Additional file 1. Comparison of mean An. gambiae s.l. and An. funestus catch by CDC light traps and indoor aspiration between CBC collections in clusters with QA teams compared to clusters where no quality-assured collections were performed. [file 12936_2023_4629_MOESM1_ESM.doc]

Additional Table 1

| Collection method | *Anopheles* species | Collectors | Efforts | Mean | RR (95% CI) |
| --- | --- | --- | --- | --- | --- |
| CDC light trap | *An. funestus* | CBC in separate houses | 7964 | 0.02 | 0.65(0.43-0.98) ^*^ |
|  |  | CBCs in same houses as QA | 2655 | 0.03 | Ref |
|  | *An. gambiae* | CBC in separate houses | 7964 | 0.09 | 0.64(0.49-0.84) ^**^ |
|  |  | CBCs in same houses as QA | 2655 | 0.24 | Ref |
|  | *An. coustani* | CBC in separate houses | 7964 | 0.002 | 0.64(0.49-0.84) ^NS^ |
|  |  | CBCs in same houses as QA | 2655 | 0.01 | Ref |
| Prokopack Aspiration | *An. funestus* | CBC in separate houses | 7878 | 0.02 | 1.75(0.98-3.11) ^NS^ |
|  |  | CBCs in same houses as QA | 2648 | 0.01 | Ref |
|  | *An. gambiae* | CBC in separate houses | 7878 | 0.08 | 0.97(0.72-1.31) ^NS^ |
|  |  | CBCs in same houses as QA | 2648 | 0.08 | Ref |
|  | *An. coustani* | CBC in separate houses | 7878 | 0.001 | 0.97(0.72-1.31) ^***^ |
|  |  | CBCs in same houses as QA | 2648 | 0.01 | Ref |
